# Iowa State Medical Society

**Published:** 1869-02

**Authors:** 


IOWA STATE MEDICAL SOCIETY.—The Seventeenth Annual Meeting of this Society will be held in the city of Des Moines, commencing at 10 o'clock, a. m., on Wednesday, the 26th of May, 1869,

Among the several committees appointed to report on various medical and surgical subjects at that time, I notice the names of Hughes, Rawson and Henry, appointed on Surgery. As chairman of that committee, I would most respectfully invite the profession, who may have anything new or interesting to offer on this subject, to report the same to me at Keokuk, or to Dr. Rawson, of Des Moines, or Dr. Henry, of Burlington, who will kindly forward to me. Parties thus forwarding will receive due credit for all matter furnished.

J. C. HUGHES, Chairman.

WE will notice in our next issue the several committees appointed by our State Medical Society, to report on various medical topics at our next annual meeting at Des Moines, in May next. Want of room prevents the notice in this number.

